# Enhanced Photovoltage Response of Hematite-X-Ferrite Interfaces (X = Cr, Mn, Co, or Ni)

**DOI:** 10.1186/s11671-017-1885-3

**Published:** 2017-02-21

**Authors:** Liang Bian, Hai-long Li, Yu-jin Li, Jia-nan Nie, Fa-qin Dong, Hai-liang Dong, Mian-xin Song, Li-sheng Wang, Tian-liang Zhou, Xiao-yan Zhang, Xin-xi Li, Lei Xie

**Affiliations:** 1 Institute of Gem and Material Technology, Hebei GEO University, Shijiazhuang, 050000 Hebei China; 2 Key Laboratory of Solid Waste Treatment and Resource Recycle, Ministry of Education, South West University of Science and Technology, Mianyang, 621010 Sichuan China; 3 Key Laboratory of Functional Materials and Devices under Special Environments, Chinese Academy of Sciences, Urumqi, 830011 Xinjiang China; 4 Department of Geology and Environmental Earth Science, Miami University, Oxford, 45056 OH USA; 5 Institute of Nuclear Physics and Chemistry, CAEP, Mianyang, 621900 Sichuan China

**Keywords:** Heterostructure, Fluorescence enhancement, Quantum dots, *Shewanella oneidensis* MR-1

## Abstract

High-fluorescent p-X-ferrites (XFe_2_O_4_; XFO; X = Fe, Cr, Mn, Co, or Ni) embedded in n-hematite (Fe_2_O_3_) surfaces were successfully fabricated via a facile bio-approach using *Shewanella oneidensis* MR-1. The results revealed that the X ions with high/low work functions modify the unpaired spin Fe^2+^–O^2−^ orbitals in the XFe_2_O_4_ lattices to become localized paired spin orbitals at the bottom of conduction band, separating the photovoltage response signals (73.36~455.16/−72.63~−32.43 meV). These (Fe_2_O_3_)–O–O–(XFe_2_O_4_) interfacial coupling behaviors at two fluorescence emission peaks (785/795 nm) are explained via calculating electron-hole effective masses (Fe_2_O_3_–FeFe_2_O_4_ 17.23 × 10^−31^ kg; Fe_2_O_3_–CoFe_2_O_4_ 3.93 × 10^−31^ kg; Fe_2_O_3_–NiFe_2_O_4_ 11.59 × 10^−31^ kg; Fe_2_O_3_–CrFe_2_O_4_ −4.2 × 10^−31^ kg; Fe_2_O_3_–MnFe_2_O_4_ −11.73 × 10^−31^ kg). Such a system could open up a new idea in the design of photovoltage response biosensors.

## Background

As a n-type semiconductor, hematite (Fe_2_O_3_) is an important semiconductor in the fields of photoluminescence and electron paramagnetic imaging due to its chemical stability and band gap (2 eV) [1]. However, poor minority charge carrier mobility (0.2 cm^2^ V^−1^ s^−1^) and ultrafast recombination of photogenerated carriers (~10 ps) limit its application as a high-photostability fluorescent material [2]. Recently, an interesting option is the conjugation of Fe_2_O_3_ with a p-type ferrite (band gaps 1.9~2.7 eV) with a lower but similar conduction band level and an appropriate valence band level [3]. It has two advantages, e.g., unpaired-paired spin change and electron-hole recombination [4]. For example, Sun et al. [5] confirmed that the electrons flow through the energy barrier between the Fe_2_O_3_ and Fe_3_O_4_ phases, according to spin-dependent tunneling mode. Higher void fraction of cubic Fe_3_O_4_ provides more transfer channels for tetrahedral ion diffusion and charge transfer. To enhance separation rate of photoinduced charge carriers, Shen et al. [6] used Mg to modify the surface photovoltage of Fe_3_O_4_/Fe_2_O_3_ heterostructured hollow nanospheres. A remarkable surface photovoltage response in UV and visible spectral region (320~570 nm) was attributed to the 2p(O^2−^)→3d(Fe^3+^) charge transfer and Fe^3+^(3d_5_) crystal field transitions of the MgFe_2_O_4_ and Fe_2_O_3_ interface. Therefore, the incorporation of the magnetic nanocrystals associated with heavy metal ion (X)-modified Fe_3_O_4_ on the Fe_2_O_3_ surface can not only improve fluorescence intensity but also recycle the fluorescence by magnetic separation under modest magnetic fields [7].

In the present work, we designed a facile approach to reduce the effect of lattice mismatch of the components, for coating directly p-ferrite on the n-hematite using *Shewanella oneidensis* MR-1 [8]. Therein, the cytochromes (OmcA, MtrC, and MtrB) and periplasmic [Fe] hydrogenases of the extracellular matrix represent the bio-reactions as follows: lactate^−^ + 4Fe^3+^ + 2H_2_O → acetat^−^ + HCO^−^ + 4Fe^2+^ + 5H^+^ [9]; X^2+^ + 2Fe(OH)_3_ → XFe_2_O_4_ + 2H_2_O + 2H^+^ [10]. Therefore, XFe_2_O_4_ particles will be directly formed from the Fe_2_O_3_ surface, creating a Fe_2_O_3_–XFe_2_O_4_ interface. The purpose of this paper is to enhance both surface photovoltage response and fluorescence, for designing new multifunctional sensor.

## Methods

Here, *S. oneidensis* MR-1 was cultured in a chemically defined minimal medium as described previously [11]. MR-1 cells were cultured aerobically on TSB (without dextrose) for 16 h at 30 °C with shaking at 100 rpm. Cells were washed twice and centrifuged at 6000 rpm for 10 min in sterile PIPES/AQDS buffer at pH 7, followed by one wash and re-suspension to ~10^9^ cells ml^−1^ in M1 medium. Anaerobically grown cells of MR-1 were added to the tubes containing the magnetite and M1 medium to obtain a final concentration of 2.3 × 10^8^ cells ml^−1^. The total volume of medium in each tube, including X(Cr, Mn, Co, or Ni)-modified goethite (FeOOH) and cells, was 250 ml. The X-modified Fe_3_O_4_ in the medium had a final concentration of 90 mM [12]. All treatment tubes were incubated in the dark at 30 °C until the end of the experiment. All treatments with anaerobically cultured cells were incubated for 45 days.

The size of the laser spot was less than 2 μm, and the acquisition time for all spectra was 20 s. Scanning electron microscopy (SEM) and energy-dispersive X-ray spectroscopy (EDS) were performed on a Cu plate with a Hitachi S-4800 field emission machine with an accelerating voltage of 5 KeV [13]. The X-ray diffraction data were collected for all Fe_2_O_3_–XFe_2_O_4_ heterostructures using an X-ray diffractometer (Bryke D8-Advance, Germany, Cu K_*α*_ radiation, *λ* = 0.154 nm, 40 kV, 40 mA) [14]. Raman scattering measurements (Labram HR evolution, Horiba Scientific, France) were conducted at room temperature under a backscattering geometric configuration using a WITec-Alpha confocal micro-Raman system [15]. The light absorption properties of the heterostructures were tested by UV-Vis diffuse reflectance spectroscopy (DRS) (Evolution 220, USA) [16]. Atomic force microscope (AFM) and Kelvin probe force microscopy (KPFM) measurements were performed on an atomic force microscope (Asylum Research MFP-3D, USA) [17]. Photoluminescence (PL) emission at room temperature was obtained at 420~900 nm with an excitation wavelength at 400 nm. The slit widths for both the excitation and emission were 5.0 nm. A fiber-based fluorescence spectrometer (USB 4000, Ocean Optics, USA) was used to record the in situ PL spectra [18].

Besides, we simulated the effective masses of electron-hole pairs, dielectric functions (*ε*), and spin-partial densities of states (spin-PDOSs) via Kramers-Kronig transform to give an insight into the electron transfer process at the atomic level and to contribute to the interpretation of experimental results from techniques such as DRS, KPFM-AFM, or PL, based on the generalized gradient-corrected Perdew-Burke-Ernzerhof functional + U (GGA-PBE) (Castep, Materials studio, Accelrys, USA) [19], where the Coulomb and screened exchange parameters (*U*, *J*) were set 5 and 1 eV, respectively. A kinetic energy cutoff of 300 eV for the electrons was used, well within the convergence of a total-energy calculation. A 2 × 2 × 2 super cell was introduced for the interstitial plane-wave, and a 5 × 5 × 5 k-point mesh for integration over the Brillouin zone.

## Results and Discussion

### Structural Characterization

**Fig. 1:**
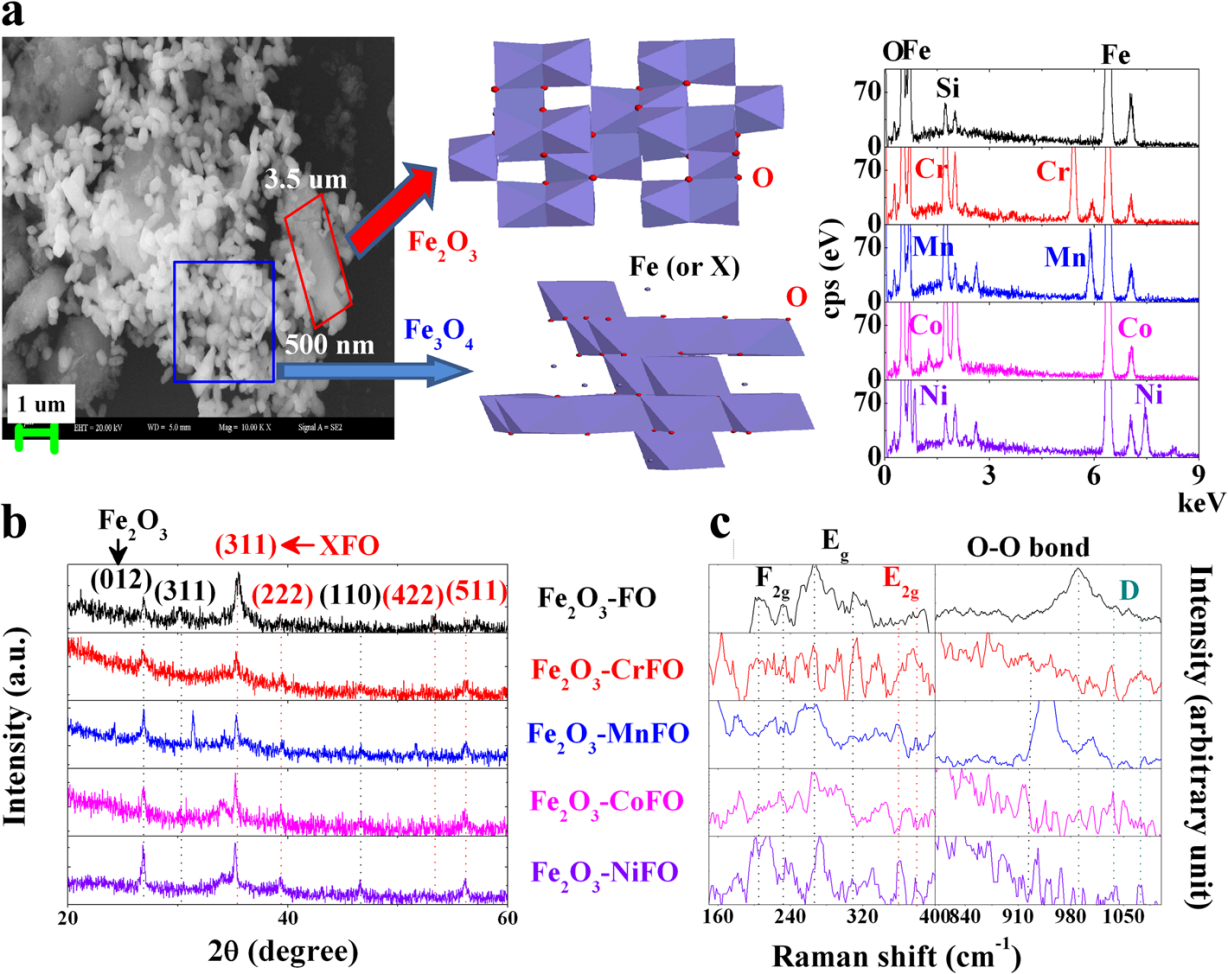
**a** SEM image of Fe_2_O_3_–Fe_3_O_4_ and EDS-SEM patterns of Fe_2_O_3_–XFe_2_O_4_. **b**, **c** show the relative XRD and Raman patterns

In Burns’ opinion [20], the reduced Fe^2+^ precipitates tend to accumulate on the extracellular polymeric substance cytochrome and [Fe] hydrogenases of *S. oneidensis* MR-1, forming the Fe_3_O_4_ phase. And H^+^ ions as the electron donors at pH6 can mediate the electron transfer between Fe^2+^ and X^2+^ to modify the Fe_3_O_4_ surface. Figure 1a confirms that X-modified Fe_3_O_4_ and Fe_2_O_3_ are coexisting, because the 500-nm rice-like Fe_3_O_4_ particles are attached onto the 3.5-μm Fe_2_O_3_ surfaces. And total mass ratios of Fe, O, and X are 26.66~57.98 wt.%, 43.61~46.65 wt.%, and 8.36~11.89 wt.%, respectively.

To verify the crystalline structure of the as-synthesized sample, typical power XRD patterns are shown in Fig. 1b. The clear diffraction peaks at 2*θ* angles (approximately 27°, 35.5°, 47°, 30.5°, 39°, 53.5°, 57.5°) can be assigned to the (012), (311), and (110) characteristic reflections of Fe_2_O_3_ (JCPDS 89-5892) and the (311), (222), (422), and (511) characteristic reflections of the cubic structure of magnetite XFe_2_O_4_ (JCPDS 19-0629) [21, 22]. The narrow and sharp peaks suggest that the obtained XFe_2_O_4_ and Fe_2_O_3_ are highly crystalline in nature, with average crystallite sizes approximately 41.32~44.21 nm and 28.78~32.79 nm at the Fe_2_O_3_–(012) and XFO–(311) planes, respectively.

To further identify the interactions of iron oxides, we used lines in Fig. 1c to depict the typical Raman spectrum of Fe_2_O_3_–XFe_2_O_4_ heterostructures. The Raman band at the F_2g_, E_g_, and E_2g_ modes reflect translational movement of the tetrahedron, symmetric bending of oxygen with respect to the metal ion, and asymmetric stretching of Fe(X) and O, respectively. These bands reflect that Fe_2_O_3_ comprises hexagonal close-packed layers of O^2−^ and that Fe^3+^ ions fill two thirds of the octahedral voids, forming a FeO_6_ octahedral layer. XFe_2_O_4_ is a normal spinel, with X^2+^ ions at tetrahedral sites and Fe^3+^ ions at octahedral sites. On another side, we can see that a broad asymmetric D peak at 915~970 cm^−1^ as the phonon scattering near the Brillouin zone boundary is attributed to the long-range (Fe_2_O_3_)–O–O–(XFe_2_O_4_) interface. These results confirm that the surface Fe_2_O_3_ has been successfully reduced as a XFe_2_O_4_ and that they are bonded to each other with the shared oxygen atoms of the octahedron-tetrahedron.

### Unpaired-Paired Spin Change in the Lattice

**Fig. 2:**
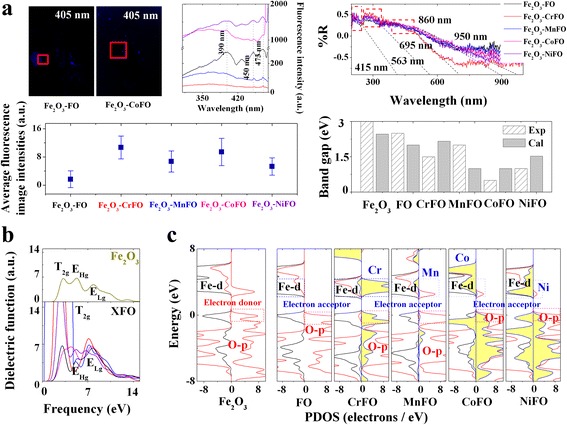
**a** Fluorescence images, average luminous intensities, and PL patterns of Fe_2_O_3_–XFe_2_O_4_ by the excitation of 405 nm. **b** reflects the DRS patterns and confrontation between experimental and calculated band gaps of Fe_2_O_3_–XFe_2_O_4_. The absorption bands were tested through the slopes of diffuse reflectance curves that the experimental and calculated band gaps are similar with each other. **c** shows the calculated dielectric functions and spin-PDOSs of Fe_2_O_3_–XFe_2_O_4_

In the literature [3], the enhanced photovoltage response performances can be attributed to intra- or inter-electronic transition in the UV and visible region. The origin of electron transfer enhancement of Fe_2_O_3_–XFe_2_O_4_ can be summarized by (i) unpaired-paired spin change in the lattice and (ii) electron-hole recombination in the interface. Figure 2a verifies that the X ions enhance the average fluorescence intensities by 3.13~6.35 multiples than that of Fe_2_O_3_–Fe_3_O_4_, where the band gap differences in Fig. 2b are close to the Fermi point, providing high electron transfer ratios. In the octahedral Fe^3+^–O^2−^ orbital at an intrinsic main fluorescence emission peak of 390 nm, the absorption bands can be attributed to two charge transfers as the oxygen-to-metal 2p(O^2−^)→3d(Fe^3+^) (left region: Fe_2_O_3_; and middle region: XFe_2_O_4_) intra-atomic transitions, reflecting the non-degeneracy (^6^A_1_)→three orbital degeneracy (T_2g_) inter-atomic transition [23]. This corresponds to the T_2g_–T_2g_ orbital degeneracy, as shown in Fig. 2c.

**Fig. 3:**
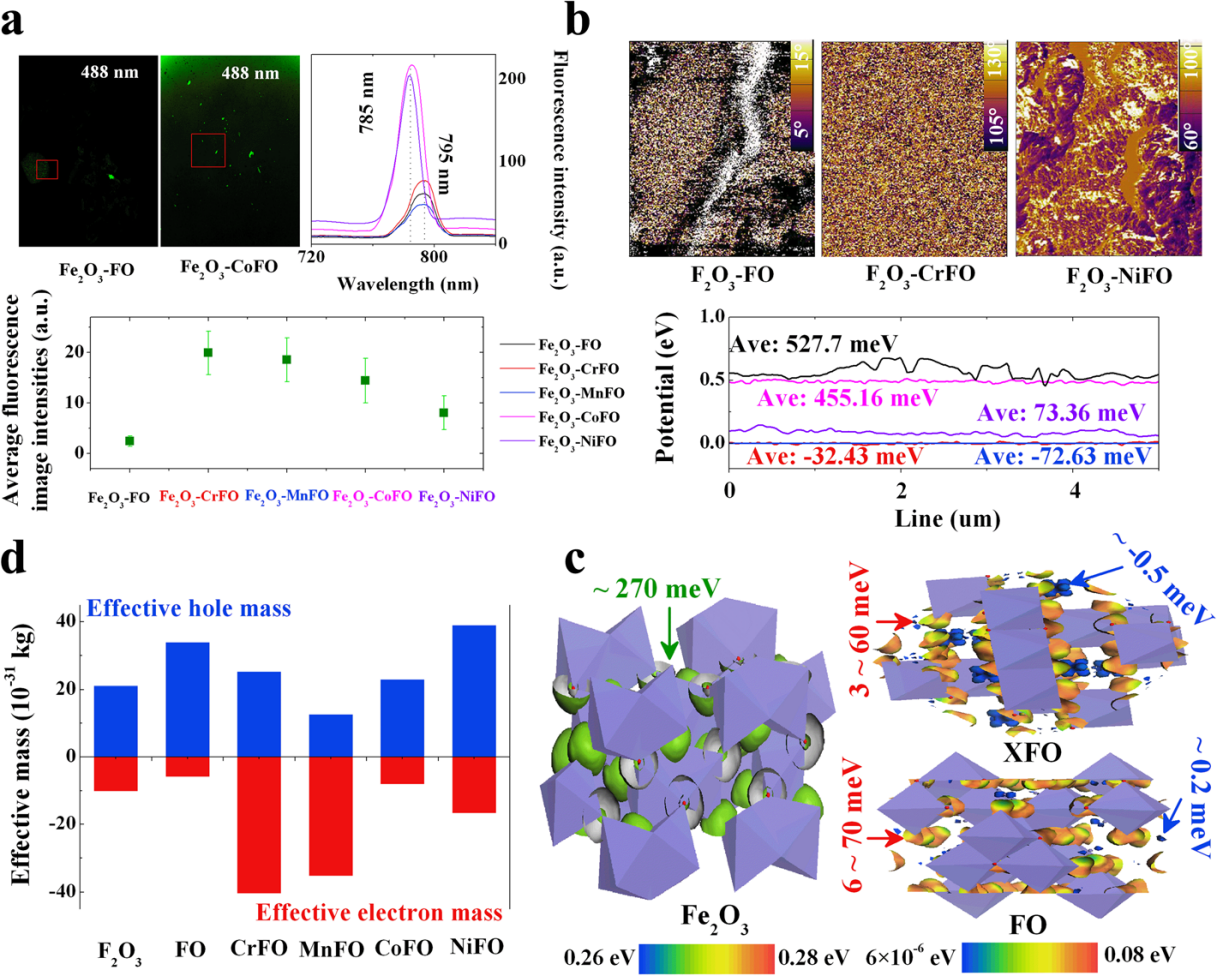
**a** Fluorescence images, average luminous intensities, and PL patterns of Fe_2_O_3_–XFe_2_O_4_ by the excitation of 488 nm. **b** reflects the surface photovoltages and KPFM phase images. The work function values of Fe_2_O_3_ and FeFe_2_O_4_ are 5.35 eV [26] and 5.52 eV [27], respectively, and the charges transfer between the different work functions of two materials for their Fermi levels to equilibrate. The corresponding theoretical surface potential images are shown in (**c**). **d** means the calculated effective masses of electron-hole pairs and surface potential illustration

On another side, the spin parity of the iron pairs can remain the same in the excited state, showing the spin-down PDOSs. To modify the electron transfer behaviors in tetrahedral Fe^2+^–O^2−^ orbitals at two broad blue fluorescence emission peaks at 450 and 473 nm, we used X^2+^ as an acceptor to occupy the tetrahedral sites at the bottom of the conduction band, and thus, the electronic transitions implying Fe^2+^(or X^2+^)–O^2−^ orbitals are known to be only at the origin of charge transfers between the bottom of the conduction band and the top of the valence band. The unpaired spin orbitals change into paired spin orbitals; therefore, the paired spin orbitals enhance the spin quantum number (*S*) to 1, changing the multiplicity of the excited state (*M* = 2*S* + 1) into 3. This unpaired-paired spin change provides more active electron gas in the Fe_2_O_3_–XFe_2_O_4_ interface to enhance the average luminous intensities as 3.31~8.18 multiples than that of Fe_2_O_3_–Fe_3_O_4_ by the excitation of 488 nm, as presented in Fig. 3a. The accelerated electron-hole recombination [24] at the near-infrared photoresponse region is beneficial for transferring long-range electrons to separate the photovoltage response signal.

### Electron-Hole Recombination in the Interface

Ultimately, we found that Fe_3_O_4_ has a significant photovoltage response red shifted to 257.7 meV from those of Fe_2_O_3_ in Fig. 3b, which allows for the efficient separation of electron-hole pairs at the Fe_2_O_3_–Fe_3_O_4_ interface, which will be higher than that (200 meV) of the reported Fe_2_O_3_–ZnFe_2_O_4_ interface [25–27]. The origin of the high/low photovoltage response region (theoretical difference data ~200 meV; experimental difference data 45~160 meV) is attributed to the surface potential difference (270 meV/3~70 meV) of Fe_2_O_3_ and XFe_2_O_4_, according to the theoretical potential images (see Fig. 3c). The photovoltage response signal in the low photovoltage response region is modified via the X–Fe–O orbital degeneracy.

To our knowledge, the crystal field theory indicates that the Fe–d orbital will split into the doubly degenerate e_g_ orbital (d_z2_ and d_x2–y2_) and triple degenerate t_2g_ orbital (d_xy_, d_yz_, and d_xz_) [28]. On the one hand, the high-energy Fe–d_x2–y2_ orbital (work function 4.5 eV) can be modified by the X–d_x2–y2_ orbital with the higher work functions (Co 4.7 eV and Ni 4.6 eV) [29]. The X ions reduce both octahedral and tetrahedral surface potentials (~0.7 and 10 meV); because of that, the down-spin Co^2+^ (or Ni^2+^)–Fe^3+^ interfacial coupling degenerates with the down-spin d_x2–y2_ orbital of Fe^2+^–3d^6^4s^2^ (Fig. 2c) at the high-energy e_Hg_ orbital. It not only enhances the positive potential direction (polarization angle 5~15° → 60~100°) but also accelerates the electron-hole recombination to create a highly effective mass hole (Fe_2_O_3_–FeFe_2_O_4_ 17.23 × 10^−31^ kg; Fe_2_O_3_–CoFe_2_O_4_ 3.93 × 10^−31^ kg; Fe_2_O_3_–NiFe_2_O_4_ 11.59 × 10^−31^ kg) (see Fig. 3d). A responsibility of the intrinsic main fluorescence emission peak blue shifts to 785 nm.

On the other hand, the Fe–O d_z2_–p_z2_ orbital is being modified by the X-d_z2_ orbital with lower work functions (Cr 4.41 eV and Mn 4.1 eV). The data curve showed that the d_z2_–d_z2_–p_z2_ orbital degeneracy creates a weak quantum well of oxygen vacancy for long-range electronic transition at the low-energy doubly degenerate e_Lg_ orbital. A part of O–2p^4^ electron is trapped in the oxygen vacancy with the deep holes present in the valence band, according to the enhanced electron effective mass (Fe_2_O_3_–CrFe_2_O_4_ −4.2 × 10^−31^ kg; Fe_2_O_3_–MnFe_2_O_4_ −11.73 × 10^−31^ kg). The down-spin p_z2_ orbital changes its direction to become an up-spin orbital, showing the negative photovoltage response signal (Cr −32.43 meV; Mn −72.63 meV). Consequently, the different photovoltage response behaviors above can be used for the design of high-mobility electronic devices and fluorescent probes for different heavy metal ion imaging, oxygen evolution catalysts [30], and biomolecules [31], etc.

## Conclusions

In summary, a novel bio-induced phase transition method for the growth of XFe_2_O_4_ embedded in a Fe_2_O_3_ is proposed here using *S. oneidensis* MR-1. We explained the mechanism of surface photovoltage response and high-photostability fluorescence. The present work demonstrates that the calculated unpaired-paired spin X^2+^–Fe^2+^–O_2-_ orbitals verify the enhanced photovoltage response signal (theoretical data ~200 meV; experimental data 45~160 meV) and separate the high/low surface potential regions (270 meV/3~70 meV) based on the calculated electron-hole effective masses. As a consequence, our works provide a reference for designing new photoluminescence and electron paramagnetic imaging biosensors. Further investigation will be focused on the electron transfer process between interfaces and heavy metal ions in an aquatic environment to garner a better understanding of the selective fluorescence probe application.
